# Supplementary material for: BioFuelDB: a database and prediction server of enzymes involved in biofuels production
Source: PeerJ. 2017 Aug 28;5:e3497. doi: 10.7717/peerj.3497 (PMC5578369; doi:10.7717/peerj.3497)
Supplement: Supplemental Information 1 [file peerj-05-3497-s001.docx]

Table S1. Distribution of enzymes and their sequences in the four application categories

| **Application Category** | **Number of Enzymes** | **Number of Sequences** |
| --- | --- | --- |
| Alcohol Production | 74 | 4968 |
| Biodiesel | 30 | 1769 |
| Fuel Cell | 27 | 1444 |
| Alternate Biofuels | 19 | 1158 |

Table S2. Distribution of enzymes and their sequences in the six EC classes

| **EC Class** | **Number of Enzymes** | **Number of Sequences** |
| --- | --- | --- |
| EC 1 | 44 | 4145 |
| EC 2 | 21 | 1118 |
| EC 3 | 42 | 1772 |
| EC 4 | 17 | 958 |
| EC 5 | 3 | 108 |
| EC 6 | 4 | 554 |

Table S3. Performance evaluation of Benz, RAPSearch and HMM on Test Dataset 1 for different EC Classes.

| **EC Classes** | **Sensitivity** | | | **Specificity** | | | **Accuracy** | | | **MCC** | | |
| --- | --- | --- | --- | --- | --- | --- | --- | --- | --- | --- | --- | --- |
|  | **Benz** | **RAP** | **HMM** | **Benz** | **RAP** | **HMM** | **Benz** | **RAP** | **HMM** | **Benz** | **RAP** | **HMM** |
| **1** | 99.38 | 94.14 | 78.88 | 99.99 | 99.84 | 98.52 | 95.16 | 86.32 | 22.47 | 0.95 | 0.86 | 0.22 |
| **2** | 99.83 | 97.79 | 93.36 | 99.98 | 99.75 | 97.25 | 96.41 | 98.01 | 70.71 | 0.96 | 0.98 | 0.71 |
| **3** | 99.98 | 98.42 | 96.35 | 99.57 | 99.62 | 84.85 | 98.8 | 99.15 | 76.94 | 0.99 | 0.99 | 0.77 |
| **4** | 96.44 | 92.49 | 25.50 | 99.96 | 99.77 | 97.81 | 98.93 | 98.45 | 59.36 | 0.99 | 0.98 | 0.59 |
| **5** | 100 | 35.14 | 97.30 | 100 | 100 | 99.78 | 100 | 100 | 45.00 | 1.00 | 1.00 | 0.45 |
| **6** | 100 | 98.42 | 91.17 | 99.98 | 100 | 99.89 | 84.06 | 81.41 | 80.34 | 0.84 | 0.81 | 0.80 |

Benz % Prediction = (14456/24009)*100 = 60.21

RAPSearch %Prediction = (22196/24009)*100 = 92.45

HMM %Prediction = (22388/24009)*100 = 93.25

Table S4. Performance evaluation of Benz, RAPSearch and HMM on Test Dataset 1 for different application categories.

| **Application Category** | **Sensitivity** | | | **Specificity** | | | **Accuracy** | | | **MCC** | | |
| --- | --- | --- | --- | --- | --- | --- | --- | --- | --- | --- | --- | --- |
|  | **Benz** | **RAPSearch** | **HMM** | **Benz** | **RAPSearch** | **HMM** | **Benz** | **RAPSearch** | **HMM** | **Benz** | **RAPSearch** | **HMM** |
| **Alcohol** | 99.48 | 95.31 | 68.21 | 99.77 | 98.09 | 93.08 | 98.27 | 96.53 | 68.11 | 0.99 | 0.93 | 0.63 |
| **Biodiesel** | 96.4 | 88.92 | 67.76 | 99.76 | 99.05 | 87.87 | 73.95 | 90.49 | 20.77 | 0.96 | 0.90 | 0.49 |
| **Fuel Cell** | 98.62 | 91.85 | 58.74 | 99.92 | 99.62 | 95.51 | 96.79 | 92.34 | 33.86 | 0.98 | 0.93 | 0.52 |
| **Other Biofuels** | 99.81 | 98.86 | 89.32 | 99.94 | 99.78 | 97.32 | 99.78 | 99.37 | 93.55 | 1 | 0.99 | 0.88 |

Table S5. Performance evaluation of Benz, RAPSearch and HMM on Test Dataset 2 for different EC Classes.

| **EC Classes** | **Sensitivity** | | | **Specificity** | | | **Accuracy** | | | **MCC** | | |
| --- | --- | --- | --- | --- | --- | --- | --- | --- | --- | --- | --- | --- |
|  | **Benz** | **RAPSearch** | **HMM** | **Benz** | **RAPSearch** | **HMM** | **Benz** | **RAPSearch** | **HMM** | **Benz** | **RAPSearch** | **HMM** |
| **1** | 99.54 | 88.14 | 72.90 | 99.76 | 97.65 | 68.03 | 98.43 | 92.62 | 53.23 | 0.99 | 0.87 | 0.39 |
| **2** | 99.93 | 87.52 | 82.97 | 99.75 | 99.43 | 89.93 | 98.61 | 96.78 | 63.61 | 0.99 | 0.91 | 0.65 |
| **3** | 100 | 76.82 | 85.66 | 99.02 | 98.01 | 90.89 | 98.86 | 96.64 | 73.18 | 0.91 | 0.76 | 0.58 |
| **4** | 99.38 | 81.89 | 76.10 | 99.94 | 99.71 | 92.77 | 99.9 | 99.43 | 73.41 | 0.99 | 0.88 | 0.57 |
| **5** | 100 | 64.36 | 70.93 | 99.99 | 99.98 | 97.97 | 100 | 100.00 | 88.36 | 0.99 | 0.79 | 0.51 |
| **6** | 100 | 85.83 | 90.50 | 99.88 | 99.83 | 95.44 | 96.03 | 88.59 | 86.77 | 0.99 | 0.91 | 0.76 |

Benz %prediction = 7292/16678 = 43.72

RAPSearch %prediction = 13468/16678 = 80.75

HMM %prediction = 23,317 /16678 = 139.81

Table S6. Performance evaluation of Benz, RAPSearch and HMM on Test Dataset 2 for different application categories.

| **Application Category** | **Sensitivity** | | | **Specificity** | | | **Accuracy** | | | **MCC** | | |
| --- | --- | --- | --- | --- | --- | --- | --- | --- | --- | --- | --- | --- |
|  | **Benz** | **RAPSearch** | **HMM** | **Benz** | **RAPSearch** | **HMM** | **Benz** | **RAPSearch** | **HMM** | **Benz** | **RAPSearch** | **HMM** |
| **Alcohol** | 99.5 | 84.33 | 70.73 | 99.43 | 94.91 | 61.00 | 98.92 | 95.97 | 64.34 | 0.98 | 0.79 | 0.31 |
| **Biodiesel** | 98.69 | 81.69 | 63.99 | 99.73 | 97.71 | 87.03 | 94.85 | 88.18 | 54.46 | 0.97 | 0.82 | 0.47 |
| **Fuel Cell** | 99.38 | 81.98 | 71.49 | 99.66 | 98.44 | 77.07 | 99.13 | 94.35 | 70.77 | 0.98 | 0.84 | 0.43 |
| **Other Biofuels** | 100 | 82.44 | 81.71 | 98.91 | 98.42 | 87.42 | 98.66 | 95.42 | 77.58 | 0.93 | 0.83 | 0.54 |
